# Tree rearrangement graphs admit paths of decreasing Robinson-Foulds distance

**Published:** 2025-12-24

**Authors:** Lena Collienne, Frederick A Matsen

**Affiliations:** 1 Computational Biology Program, Fred Hutchinson Cancer Research Center, Seattle, Washington, USA; 2 Howard Hughes Medical Institute, Fred Hutchinson Cancer Research Center, Seattle, Washington, USA; 3 Department of Statistics, University of Washington, Seattle, USA; 4 Department of Genome Sciences, University of Washington, Seattle, USA

**Keywords:** Phylogenetics, treespace, tree rearrangements, tree distances

## Abstract

Tree rearrangements such as Nearest Neighbor Interchange (NNI) and Subtree Prune and Regraft (SPR) are commonly used to explore phylogenetic treespace. Computing distances based on them, however, is often intractable, so the efficiently computable Robinson-Foulds (RF) distance is used in practice. We investigate how the RF distance behaves along paths in the NNI and SPR graphs, where trees are nodes, edges represent single rearrangements. We show that any two trees are connected by a path along which the RF distance to the target decreases monotonically in the NNI graph and strictly in the SPR graph; we also exhibit trees for which no strictly decreasing NNI path exists.

## 1 Introduction

Phylogenetic trees are complex objects that can be viewed from many perspectives (St. John, 2017). One perspective considers a tree as a collection of bipartitions: statements about which leaves are separated from other leaves. These bipartitions are called splits; trees are in one-to-one correspondence with their sets of splits. One can turn this perspective into a distance between phylogenetic trees by considering the size of the symmetric difference between their split collections. This distance, formally defined below, is called the Robinson-Foulds (RF) distance.

Another perspective on phylogenetic trees is as an organized collection of subtrees. A natural operation on a tree from this perspective is to separate a subtree from one location and move it to another location in the tree. If that movement just swaps subtrees across an internal edge, this is called a Nearest Neighbor Interchange (NNI). If the movement reattaches the subtree on an arbitrary edge, this is called a Subtree Prune and Regraft (SPR). One can build a graph such that the vertices of the graph are phylogenetic trees, and adjacency in the graph is determined by whether one tree can be transformed to another by one of these operations.

The corresponding graph distance for the NNI (resp. SPR) adjacency is called the NNI (resp. SPR) distance. For both NNI and SPR it has been shown that computing the distance between two trees is *NP*-hard (DasGupta et al., 2000; Bordewich and Semple, 2005b; Hickey et al., 2008). In contrast, the Robinson-Foulds (RF) distance is computable in linear time.

The intractable rearrangement-based distances are generally considered preferable to the RF distance. First, these distances have biological interpretations in terms of hybridizations (Bordewich and Semple, 2005a, 2007). Second, these distances correspond to the optimization steps of popular maximum likelihood methods and proposal moves for Markov-Chain Monte Carlo (MCMC) Bayesian phylogenetic algorithms. However, as stated above, these distances are hard to compute.

Consequently, exploration of large collections of trees (e.g. Gao et al., 2025, analyzing MCMC chains) often uses both RF and SPR distances. RF can be computed for all pairs in these collections. SPR is intractable for large datasets, so researchers resort to approximations and subsampling. The conclusions drawn from RF and SPR distances often differ substantially (Brusselmans et al., 2024; Gao et al., 2025).

Hence, we are motivated to understand the relationship between RF and tree rearrangement distances. Previous work has shown that the RF distance is not robust with respect to the SPR rearrangement in the sense that trees with a small SPR distance can be very distant under Robinson-Foulds. For example, a single SPR move can connect two trees with maximum RF distance, if the pruned subtree contains just one leaf that gets reattached on the opposite end of a tree (Böcker et al., 2013). On the other hand, because NNI moves are more local in a tree and only change one edge, the NNI distance between two trees is at most twice as large as their RF distance (Steel, 2016).

Despite these advances, our knowledge of the connection between these distances remains incomplete. As described above, the NNI (resp. SPR) graph is the graph formed with trees as nodes and edges representing NNI (resp. SPR) moves. The work described in the previous paragraph compares shortest paths in these graphs to the RF distance, and shows that these can be in conflict. However, it does not address the question of how the RF distance changes on arbitrary paths through these graphs.

Specifically, given a pair of trees T and R, is there a path $\left[T≅T_{0},T_{1},\ldots ,T_{k}≅\right.R]$ between them in NNI or SPR graph such that the RF distance between $T_{i}$ and R is monotonically decreasing? A negative answer would argue that these measures are fundamentally at odds. This question was posed as an open problem by Nick Goldman at the “Algorithmic Advances and Implementation Challenges: Developing Practical Tools for Phylogenetic Inference” workshop at the Institute for Computational and Experimental Research in Mathematics in Providence, RI in November 2024.

In this paper, we answer this question in the affirmative. Specifically, we show that any two trees are connected by a path in the NNI graph on which the RF distance to the target tree is monotonically decreasing along the path; we also exhibit a pair of trees for which it cannot be strictly decreasing. In the SPR graph, however, we can always find a path with strictly decreasing RF distance to the final tree.

## 2 Definitions

A *phylogenetic tree* is a tree with leaves bijectively labeled by elements of a set X. For simplicity, we refer to phylogenetic trees as *trees*. Unless otherwise stated, we assume that trees are unrooted and binary, i.e. all internal nodes are of degree three. Two phylogenetic trees are isomorphic if they are isomorphic as graphs and the isomorphism preserves leaf labels. For isomorphic trees T and R we write T≅R.

A split A∣B of a tree T with leaf labels in X is a bipartition of X so that cutting an edge e in T results in two connected components. We also say that e *induces* the split A∣B. The split set Γ(T) is the set of all splits of T, and it uniquely identifies T (Robinson and Foulds, 1981). We define the *Robinson-Foulds* (RF) distance between two trees T and R as the symmetric difference of the size of their split sets: $d_{RF}(T,R)=|\Gamma (T)\Delta \Gamma (R)|$. A split A∣B is called *trivial* if either A or B contains only one element, otherwise it is called *non-trivial*. For a subset A⊆X, the subtree of T induced by A is the smallest connected subgraph of T containing all leaves in A, with degree-2 vertices suppressed.

In this paper we focus on two tree rearrangement operations: Nearest Neighbor Interchange and Subtree Prune and Regraft. Trees T and R are connected by a *Nearest Neighbor Interchange* (NNI) move if there is an edge e in T and an edge f in R so that shrinking e and f to a single node results in isomorphic (non-binary) trees. A *Subtree Prune and Regraft* SPR move on a tree T begins by removing an edge e to receive two connected components $T_{1}$ and $T_{2}$, each of which have one degree-2 node. Then a new node *v* is added on an edge f in $T_{2}$, which gets connected to the degree-2 node in $T_{1}$ by a new edge, and the remaining degree-2 node in the resulting graph gets suppressed.

Tree rearrangements define a graph where nodes represent trees on n leaves that are connected by an edge if the corresponding trees are connected by a tree rearrangement. We call these graphs NNI graph and SPR graph. A *path* between trees T and R is a sequence [$T≅T_{0},T_{1},\ldots ,T_{k-1},T_{k}=R$] of trees so that $T_{i}$ and $T_{i+1}$ are connected by a tree rearrangement. The *distance* between trees T and R in NNI or SPR graph is the length of a shortest path connecting T and R, or in other words, the minimum number of tree rearrangements required to transform one tree into the other.

## 3 Robinson-Foulds distance along tree rearrangement paths

In this section we show that given a source and a target tree, we can find paths in the NNI graph along which the Robinson-Foulds distance to the target tree decreases monotonically. For the SPR graph we can even show the existence of paths with strictly decreasing RF distance.

### Paths in the NNI graph

In the following, we show there is a path between any two trees T and R in NNI graph so that the RF distance to R decreases along this path from T and R.

#### Theorem 1.

*For arbitrary trees* T *and* R *there is a path* $\left[T_{0}≅T,T_{1},\ldots ,T_{k}≅\;\right.R]$ *in* NNI *graph with* $d_{RF}\left(T_{i+1},R\right)\leq d_{RF}\left(T_{i},R\right)$ *for all* i=0,…,k-1.

*Proof.* We prove this theorem by induction on the number of leaves of T and R. For the induction basis we consider trees T and R with four leaves and therefore one internal edge. With just one internal edge, the RF distance is either zero or two. If $d_{RF}\left(T,R\right)=0,T$ and R are isomorphic and the theorem is true with the path consisting of just one tree T≅R. If $d_{RF}\left(T,R\right)=2,\;T$ and R are not isomorphic. Because all trees on four leaves are connected by an NNI move, the path [$T_{0}≅T,T_{1}≅R$] fulfills the conditions of the theorem.

For the induction step we assume that the theorem is true for all pairs of trees with less than or equal to n leaves. Let T and R be trees on n+1 leaves. We distinguish the case (i) that T and R share at least one non-trivial split from the case (ii) that they have no splits in common, i.e. they have maximum RF distance.

**Figure 1:**
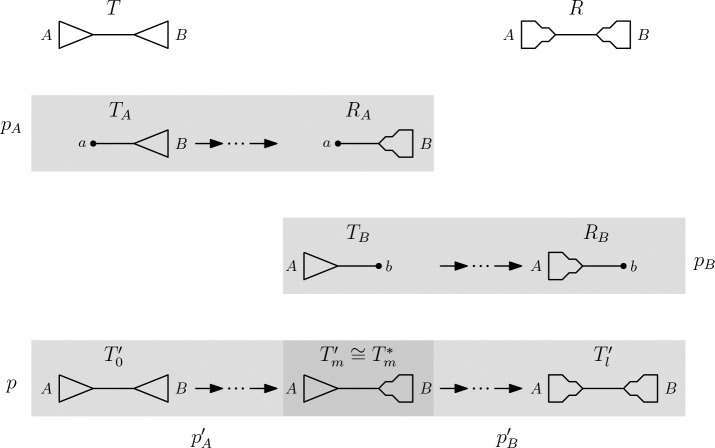
Trees T and R in the top row, paths $p_{A}$ and $p_{B}$ in the middle rows, and the concatenation of paths $p_{A}^{\prime }$ and $p_{B}^{\prime }$ in the bottom row. All subtrees induced by A (or B) are isomorphic if they are illustrated in the same way (triangles as in T and rugged triangles as in R).

**Figure 2:**
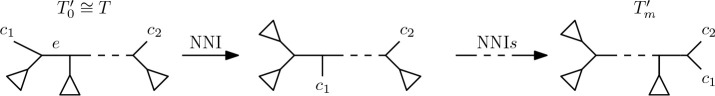
Trees $T_{0}^{\prime }≅T$ and $T_{m}^{\prime }$ and connected by a sequence of NNI moves to build the cherry ($c_{1},c_{2}$) as in case (ii) of Theorem 1.

- Let T and R be trees that both have an internal edge inducing the same non-trivial split A∣B. Let $T_{A}$ and $T_{B}$ be the trees that result from replacing the subtrees induced by A and B with leaves a and b, respectively, and let $R_{A}$ and $R_{B}$ the trees resulting from the same subtree replacements in R. These trees as well as the paths described in the following are shown in Figure 1. $T_{A}$ and $R_{A}$ have leaf set (X\A)∪{a} and $T_{B}$ and $R_{B}$ have leaf set (X\B)∪{b}. Since A∣B is a non-trivial split, we have |A|>1 and |B|>1 and therefore, $T_{A}$, $R_{A},\;T_{B}$, and $R_{B}$ all have less than n+1 leaves. Applying the induction hypothesis gives us paths $p_{A}$ from $T_{A}$ to $R_{A}$ and $p_{B}$ from $T_{B}$ to $R_{B}$ that fulfills the criteria of the theorem.
  We now use $p_{A}$ and $p_{B}$ to construct a path from T to R. By replacing the leaf a in every tree on $p_{A}$ by the subtree of T that is induced by A we receive a Path $p_{A}^{\prime }=\left[T_{0}^{\prime },T_{1}^{\prime },\ldots ,T_{m}^{\prime }\right]$. This replaces a in the split sets of all trees along $p_{A}$ with A, which implies that the size of the symmetric differences between the trees along $p_{A}$ and the final tree is the same as those of the trees along $p_{A}^{\prime }$ and the corresponding final tree. $p_{A}^{\prime }$ therefore inherits the property of a decreasing Robinson-Foulds distance to its final tree from the original path $p_{A}$. Similarly, replacing the leaf b in all trees on $p_{B}$ by the subtree of R induced by B gives a path $p_{B}^{\prime }=\left[T_{m}^{*},T_{m+1}^{\prime },\ldots ,T_{l}^{\prime }\right]$ on which the Robinson-Foulds distance to $T_{l}^{\prime }$ is monotonically decreasing.
  We now argue that $T_{m}^{\prime }$ and $T_{m}^{*}$ are isomorphic by showing that their split sets are identical. By definition, A∣B is a split in both $T_{m}^{\prime }$ and $T_{m}^{*}$. Let X∣Y be a split in $T_{m}^{\prime }$. We consider three cases to show that X∣Y is a split in $T_{m}^{*}$, too: X=A, X⊂A, or X⊈A. For X=A, it is obvious that X∣Y is a split in $T_{m}^{*}$. By definition of $T_{m}^{*}$, the subtree induced by A is isomorphic to the subtree induced by A in T, which implies that the same is true for X⊂A. Therefore, X∣Y is a split in $T_{m}^{*}$. If X⊈A, it must be Y⊆A, as A∣B is a split in $T_{m}^{\prime }$, and we can apply the previous cases to Y to see that X∣Y is a split in $T_{m}^{*}$.
  We can therefore concatenate $p_{A}^{\prime }$ and $p_{B}^{\prime }$ to receive a path
$$p=\left[T≅T_{0}^{\prime },T_{1}^{\prime },\ldots ,T_{m}^{\prime }≅T_{m}^{*},T_{m+1}^{\prime },\ldots ,T_{l}^{\prime }≅R\right]$$
  from T to R. By the construction of p, the Robinson-Foulds distance of trees along p to R is decreasing along the path. Therefore, p is a path between T and R as described in the theorem.
- Let T and R be trees that share no split. Let $\left(c_{1},c_{2}\right)$ be a cherry in R: a pair of leaves $c_{1}$ and $c_{2}$ that are adjacent to the same internal node. By definition, this cherry cannot appear in T. Then there is a sequence of NNI moves that moves $c_{1}$ along the tree towards $c_{2}$, resulting in a sequence $T≅T_{0}^{\prime },T_{1}^{\prime },\ldots ,T_{m}^{\prime }$ of trees. To construct such a sequence, one starts at the tree T and performs an NNI move on the edge e that is the second edge on the path from $c_{1}$ to $c_{2}$ in T, and swaps $c_{2}$ with a subtree on the other side on e as displayed in Figure 2.
  By repeating this procedure, we eventually arrive at a tree $T_{m}^{\prime }$ where $c_{1}$ and $c_{2}$ are siblings. Thus $d_{RF}\left(T_{m}^{\prime },R\right)<d_{RF}(T,R)$. Let i be the smallest index such that $d_{RF}\left(T_{i}^{\prime },R\right)<d_{RF}(T,R)$. Because the Robinson-Foulds distance between T and R is already at its maximum, we have $d_{RF}\left(T_{0}^{\prime },R\right)=d_{RF}\left(T_{1}^{\prime },R\right)=\cdots =d_{RF}\left(T_{i-1}^{\prime },R\right)>d_{RF}\left(T_{i}^{\prime },R\right)$. A tree $T_{i}^{\prime }$ with this property is reached at the latest when the cherry ($c_{1},c_{2}$) is constructed. Applying case (i) to $T_{i}^{\prime }$ and R gives us a path with properties stated in the theorem.

It is important to allow adjacent trees on the NNI path to have the same RF distance to R in Theorem 1. We show in Theorem 2 that we cannot assume strictly decreasing RF distances along an NNI path.

#### Theorem 2.

*There are trees* T *and* R *for which no path* $\left[T≅T_{0},T_{1},\ldots ,T_{k}≅\right.R]$ *exists in* NNI *graph with* $d_{RF}\left(T_{i+1},R\right)<d_{RF}\left(T_{i},R\right)$.

**Figure 3:**
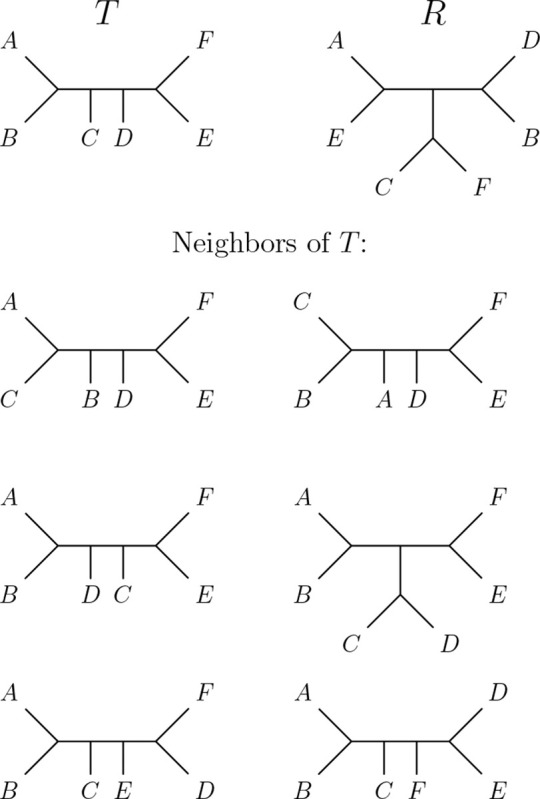
Trees T and R of counterexample for proof of Theorem 2.

*Proof.* Let T and R be the trees in the top row in Figure 3. Then $d_{RF}(T,R)=6$, as all three internal edges induce different splits in T and R. There is no NNI move on T that results in an edge representing one of the splits of R, so every NNI neighbor of T also has distance 6 to R (see full list of neighbors in Figure 3). Therefore, no path as described in the theorem can exist.

### Paths in the SPR graph

SPR moves are a generalization of NNI moves, which means that every NNI move is an SPR move (Allen and Steel, 2001). By Theorem 1 there is always a path in SPR on which the Robinson-Foulds distance to the final tree decreases monotonically. In this section we show an even stronger result: There is a path between any two trees in the SPR graph along which the RF distance to the destination tree is strictly decreasing.

#### Theorem 3.

*For arbitrary trees* T *and* R *there is a path* $\left[T≅T_{0},T_{1},\ldots ,T_{k}≅\;\right.R]$ *in* SPR *graph with* $d_{RF}\left(T_{i+1},R\right)<d_{RF}\left(T_{i},R\right)$ *for all* i=0,…,k-1.

*Proof.* We assume that there is at least one split in T that is not present in R, as otherwise the trees are isomorphic and the path consists of one tree only. The size of a split A∣B is min(|A|,|B|). Let A∣B be a non-trivial split of minimum size in T that is not a split in R. Since T is binary and A∣B is a non-trivial split in T, there must be sets $A_{1}$ and $A_{2}$ that are induced by subtrees in T so that $A_{1}\cup A_{2}=A$. By the minimality assumption on A∣B, there must also be subtrees in R that induce $A_{1}$ and $A_{2}$.

Let $R_{A_{1}}$ be the subtree of R induced by $A_{1}$, and $R_{A_{2}}$ the subtree induced by $A_{2}$. We can perform an SPR move on R that prunes $R_{A_{1}}$ and reattaches it on the edge connecting $R_{A_{2}}$ with the rest of the tree. This creates an edge that induces the split A∣B. Let $R^{\prime }$ be the tree resulting from this SPR move.

We now consider the differences in the split sets of R and $R^{\prime }$ when comparing to T. By our assumption that A∣B is not a split in R, there is a path of edges between the subtrees $R_{A_{1}}$ and $R_{A_{2}}$ in R where each edge induces a split of the form $\left(A_{1}\cup X\right)|\left(A_{2}\cup Y\right)$ for some X,Y≠∅. These are the only splits that change between R and $R^{\prime }$ when moving the subtree $R_{A_{2}}$, and we denote the set of these splits by S.

Since $A\left|B=\left(A_{1}\cup A_{2}\right)\right|B$ is a split in T, no splits of S are present in T. It follows that S is a subset of the splits of R, but not of T. Therefore, S is a subset of the symmetric difference of the split sets of T and R. This means that changing ony edges in inducing splits in S between R and $R^{\prime }$ cannot increase the RF distance to T. Since the SPR move between R and $R^{\prime }$ transforms one split of S to become A∣B and changes only splits within S, $R^{\prime }$ has at least one more split in common with T than R does. Therefore, $d_{RF}\left(R^{\prime },T\right)<d_{RF}(R,T)$.

We can repeatedly apply this argument to the next smallest split in T that is not present in $R^{\prime }$ and thereby build a path from R to T on which the RF distance strictly decreases in every step.

Tree Bisection and Reconnection (TBR) is another tree rearrangement operation and generalizes SPR. It starts by removing an edge and suppressing the resulting degree-2 nodes, we receive two connected components. Two arbitrary edges are chosen, one in either of the two components, and a new node is introduced on each of these edges. By connecting these new nodes, we finish the TBR move and receive a binary phylogenetic tree. As all SPR moves are TBR moves (Allen and Steel, 2001), we get the following corollary.

#### Corollary 1.

*For arbitrary trees* T *and* R *there is a path* $\left[T≅T_{0},T_{1},\ldots ,T_{k}≅\right.R]$ *in* TBR *graph with* $d_{RF}\left(T_{i+1},R\right)<d_{RF}\left(T_{i},R\right)$ *for all* i=0,…,k-1.

## 4 Discussion

In this paper we show that for any two trees T and R there is a path in the NNI graph along which the Robinson-Foulds distance to R decreases monotonically. We also give an example to show that we cannot guarantee that the RF distance will strictly decrease along an NNI path. In the SPR graph, however, there is a path with strictly decreasing RF distance to the destination tree. These results give some insight into the relationship of Robinson-Foulds and tree rearrangement based distances.

The constructive proof of Theorem 3 can be used to generate paths along which the RF distance to the destination tree decreases. These paths are generally not shortest paths in the SPR graph, but they do preserve all shared splits, in contrast to shortest SPR paths, which may break shared splits (Whidden and Matsen, 2019). Understanding the relationship between shortest paths and paths with monotonically decreasing RF distances remains an open direction for future research.

## Figures and Tables

**Figure 4:**
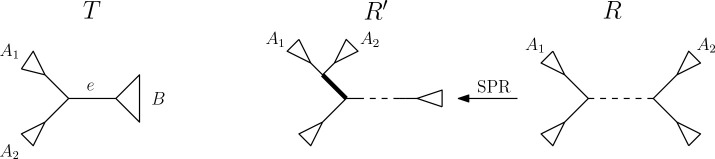
Trees T and R and $R^{\prime }$ in the proof of Theorem 3. In T on the left, edge e induces the split A∣B. In R on the right and $R^{\prime }$, connected by an SPR move to R that moves the subtree induced by $A_{2}$, in the middle. The dashed line in R and $R^{\prime }$ represents a section of the tree that might contain more leaves and edges. The dashed path in R contains all S that induce splits not present in T. The edge highlighted in bold in $R^{\prime }$ induces the split A∣B.

## Data Availability

Data sharing is not applicable to this article as no new data were created or analyzed in this study.
